# Randomised controlled trial of the effects of L-ornithine on stress markers and sleep quality in healthy workers

**DOI:** 10.1186/1475-2891-13-53

**Published:** 2014-06-03

**Authors:** Mika Miyake, Takayoshi Kirisako, Takeshi Kokubo, Yutaka Miura, Koji Morishita, Hisayoshi Okamura, Akira Tsuda

**Affiliations:** 1 Research Laboratories for Health Science & Food technologies, Research & Development Division, Kirin Co., Ltd., 1-13-5, Fukuura, Kanazawa-ku, Yokohama, Kanagawa 236-0004, Japan; 2 Healthcare Products Development Center, Kyowa Hakko Bio Co., Ltd., 2, Miyukigaoka, Tsukuba, Ibaraki, 305–0841, Japan; 3 Cognitive and molecular of Brain disease, Kurume University, 67, Asahi-machi, Kurume, Fukuoka 830-0011, Japan; 4 Department of Psychology, Kurume University, 1635, Miimachi, Kurume, Fukuoka 839-8502, Japan

**Keywords:** L-ornithine supplementation, Stress and sleep, Clinical trial

## Abstract

**Background:**

L-ornithine is a non-essential, non-protein amino acid. Although L-ornithine is contained in various foods, the amount is usually small.

Recently, studies have shown that orally administered L-ornithine reduced the stress response in animals.

From these findings, we speculated that L-ornithine may play a role in the relieve of stress and improve sleep and fatigue symptoms in humans. Through a randomised, double-blind, placebo-controlled clinical study, we asked if L-ornithine could be beneficial to stress and sleep in healthy workers.

**Method:**

Fifty-two apparently healthy Japanese adults who had previously felt slight stress as well as fatigue were recruited to be study participants and were randomly divided into either the L-ornithine (400 mg/day) or placebo group. They orally consumed the respective test substance every day for 8 weeks. Serum was collected for the assessment of cortisol and dehydroepiandrosterone-sulphate (DHEA-S). Perceived mood and quality of sleep were measured by the Profile of Mood States (POMS), Athens Insomnia Scale (AIS), and Ogri-Shirakawa-Azumi sleep inventory MA version (OSA-MA).

**Results:**

Serum cortisol levels and the cortisol/DHEA-S ratio were significantly decreased in the L-ornithine group in comparison with the placebo group. Also, anger was reduced and perceived sleep quality was improved in the L-ornithine group.

**Conclusion:**

L-ornithine supplementation has the potential to relieve stress and improve sleep quality related to fatigue, both objectively and subjectively.

## Background

Sleep is both necessary and universal. Like eating and drinking, without it we will eventually die. Sleep is affected by various psychosocial factors, stress, and the daily routine, and it similarly affects all areas of life. Sleep is reported to be an important mediator of health through the autonomic nervous and immune systems and endocrine function [1-5].

Sleep and fatigue often correlate highly in cross-sectional studies, and reduced sleep duration involves the gradual accumulation of sleepiness/fatigue [6]. Fatigue is generally considered to be important in daily life as it is closely related to quality of life and well being. Chronic or accumulated fatigue plays a role in an individual’s performance of various functions. At worst, long-term accumulated fatigue can lead to *karoshi* (death from overwork) [7]. Nevertheless, fatigue is a complex multidimensional concept that involves physical and psychosocial aspects. Psychological fatigue is closely associated with stress [8,9].

Stress has been shown to induce a physiological response that is mediated by the hypothalamic-pituitary-adrenal (HPA) axis leading to the release of cortisol in humans and corticosterone in mice [10]. Cortisol has long been used as a marker of stress [11]. Dehydroepiandrosterone (DHEA) and its sulfate ester (DHEA-S) are the most abundant adrenal androgens. They are produced as precursors to the sex hormones estradiol and testosterone from adrenal glands and their serum levels decrease with age [12-14]. DHEA-S has cortisol-lowering effects [15] and may attenuate the adverse health effects of hypercortisolism [16]. DHEA-to-cortisol ratios in serum and saliva are likely to be more reliable than concentrations of either hormone alone, with lower morning ratios seen in depression [17-19].

Recently it was reported that orally administered L-ornithine reduced restraint stress-induced activation of the HPA axis in mice accompanied by a reduction in the serum corticosterone concentration [20]. It was shown that an intracerebroventricular (i.c.v.) injection of L-ornithine attenuated the stress response in neonatal chicks. These actions were suggested to be mediated by the gamma-aminobutyric acid (GABA) receptor.

From these findings, we speculated that L-ornithine may play a direct role in the central nervous system, relieve stress and improve sleep and fatigue symptoms in humans. L-ornithine is a non-essential, non-protein amino acid. Although L-ornithine is contained in various foods, the amount is usually small. Since ancient times, corbicula, a genus of the clam, has been considered to be good for the liver. They were found to contain 159.9 mg L-ornithine per 100 g of the extract [21], which is high compared with other foods [22] but still a relatively small amount.

Orally administered L-ornithine is transferred to the portal vein from the intestines and delivered to various tissues, such as the liver, kidney and muscle [23]. In liver, L-ornithine plays a central role in the urea cycle which converts ammonia to urea [24]. L-ornithine administration has been known to enhance detoxification of ammonia in the liver [25].

Using a randomised, double-blind, placebo-controlled clinical trial, we evaluated the effect of long-term ingestion of L-ornithine on stress-related markers in serum and subjective feelings associated with stress and sleep in study subjects who indicated feelings of slight fatigue.

## Methods

### Study design

This study was a randomised, double-blind and placebo-controlled trial. Subjects were randomly allocated to either the L-ornithine group or placebo group.

Most previous studies have evaluated the short term effect of L-ornithine supplementation on healthy volunteers [26,27]; we investigated the long term effect of L-ornithine supplementation on healthy volunteers. Eight weeks was chosen as the study period after consideration of the seasonal effect and the volunteer's burden.

Subjects ingested either L-ornithine or placebo capsules before going to bed every day for 8 weeks. Blood was collected four times during this clinical trial: before supplementation (0w), corresponding to the screening evaluation; 2 weeks (2w) and 4 weeks (4w) after taking the supplement; and at the end of the trial (8w).

We used the following three questionnaires to evaluate perceived stress, sleep quality and mood state: POMS [28], Athens Insomnia Scale (AIS) [29] and Ogri-Shirakawa-Azumi sleep inventory MA version (OSA-MA) [30]. The POMS questionnaire is a well established, factor-analytically derived measure of psychological distress, such as mood, for which high levels of reliability and validity have been documented. We used the short-form POMS, which consists of thirty adjectives rated on a 0–4 scale that can be consolidated into six mood scales: “tension-anxiety”, “depression-dejection”, “anger-hostility”, “vigor”, “fatigue” and “confusion”. The T score of the POMS questionnaire was calculated using the standard method [28], showing that higher T scores represent high levels of distress, with the exception of “vigor”. This questionnaire was completed by each study participant at 0, 2, 4, 6 and 8 weeks.

The AIS is a useful tool to assess the existence of insomnia. This self-administered psychometric instrument consists of eight items: difficulty with sleep induction, awakening during the night, early morning awakening, total sleep time, overall quality of sleep, problems with sense of well-being, and functioning, and sleepiness during the day. Each item was rated on a scale of 0 to 3, with 0 corresponding to “no problem at all” and 3 indicating “a very serious problem”. Thus, the total AIS score ranges from 0 (denoting absence of any sleep-related problem) to 24 (representing the most severe degree of insomnia). Volunteers completed this questionnaire at 0, 4, and 8 weeks.

The OSA sleep inventory is popularly used for evaluation of sleep quality in Japan. The MA version is more useful for middle-aged and old-aged people and consists of sixteen adjectives with responses rated on a 0–4 scale that can be consolidated into five factors: “sleepiness on rising”, “initiation and maintenance of sleep”, “frequent dreaming”, “refreshing” and “sleep length”. The OSA-MA score was calculated using an MS-Excel sheet [30], with higher scores indicating a good quality of sleep: this questionnaire was completed weekly for 8 weeks.

The present study was conducted according to the guidelines laid down in the Declaration of Helsinki and all procedures involving human subjects were approved by the local ethics committee of Medical Corporation, Akihabara Medical Clinic. Written informed consent was obtained from all participants.

### Study population

Fifty-two apparently healthy Japanese individuals participated in this study. The volunteers ranged in age from 30 to 60 years (male: female ratio, 2:3), and had full-time jobs, excluding those who engaged in shift work or physical work, such as carpenter or delivery person, and irregular work schedules. Subjects were selected based on their POMS questionnaire score, which should be above 50 in T scores of “fatigue” and below 50 in T scores of “vigor”.

Smokers, pregnant or lactating women, or persons who habitually took L-ornithine, medication or supplements to improve stress, fatigue or sleep were excluded from this study. Also excluded were those with a past history of diabetes, hepatic disease, renal disease, hypertension, ischemic heart disease or abnormal glucose tolerance.

### Test substance

L-Ornithine monohydrochloride and microcrystalline cellulose (FD-301) were purchased from Kyowa Hakko Bio (Tokyo, Japan) and Asahi Kasei Chemicals (Tokyo, Japan), respectively. Two kinds of small hard capsules were prepared, with one type of capsule containing 500 mg of L-ornithine monohydrochloride (400 mg L-ornithine) and 160 mg of microcrystalline cellulose per 2 capsules and the other (placebo) containing 560 mg of microcrystalline cellulose per 2 capsules.

### Measurement of serum cortisol and DHEA-S

Participants were instructed not to eat breakfast after 8:00 am and abstain from smoking and caffeinated drinks prior to blood sampling. Blood samples were collected by venipuncture from all participants between 11:00 and 13:00. Serum cortisol and DHEAS levels were analyzed by commercial laboratories (BML Inc., Tokyo, Japan).

### Statistical analysis

Values are presented as the mean ± standard error (SEM). Two-way analysis of variance was used to evaluate the significance of differences between the placebo and L-ornithine groups followed by pairwise comparisons and unpaired *t* tests. *P* values less than 0.05 were considered to be statistically significant.

## Results

### Background information or demographic data

There were no statistical differences between the two groups at baseline (Table 1). During the 8 study weeks of the study, no adverse events were observed.

**Table 1:** Characteristics of subjects at 0 week

		**Placebo group**		**Ornithine group**	
Men (n)		11		10	
Women (n)		15		16	
		Average	SE	Average	SE
Age (years)		43.38	1.56	43.31	1.46
POMS	Tension-anxiety	63.35	1.66	65.23	1.99
	Depression-dejection	65.69	2.32	64.19	2.26
	Anger-hostility	61.85	2.29	63.31	2.28
	Vigor	35.62	1.05	36.88	1.12
	Fatigue	66.96	1.26	68.77	1.28
	Confusion	68.31	1.85	69.77	1.92
AIS		7.96	0.61	8.54	0.54
OSA	Sleepiness on rising	9.37	0.87	7.98	1.10
	Initiation and maintenance of sleep	15.21	1.34	12.12	1.17
	Frequent dreaming	21.73	1.62	23.04	1.40
	Refreshing	10.92	1.09	10.27	0.88
	Sleep length	13.31	1.11	14.71	1.40
Cortisol (μM)		0.21	0.02	0.23	0.01
DHEA-S (μM)		4.25	0.32	4.72	0.44
Cortisol/DHEA-S × 100		5.22	0.40	5.67	0.56

Most of the study subjects were general office workers with 24 out of 26 participants in the placebo group, and 23 out of 26 participants in the L-ornithine group.

### L-Ornithine supplementation improved mood state in study participants

**Figure 1:**
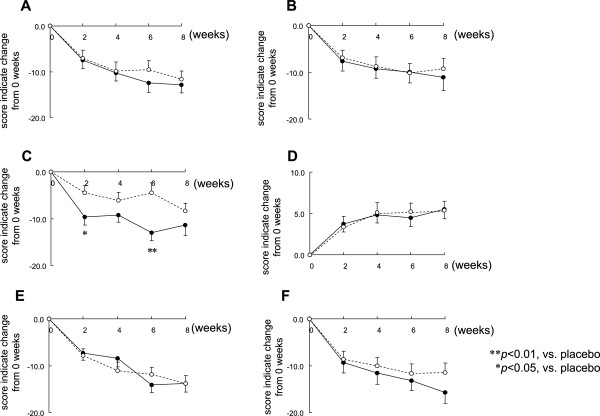
**Effect of L-ornithine supplementation on POMS.** Means of the change from 0 weeks of each POMS score (**A**, tension-anxiety; **B**, depression-dejection; **C**, anger-hostility; **D**, vigor; **E**, fatigue; **F**, confusion) to 2, 4, 6 and 8 weeks: mean ± SE. White circles (○) indicate the placebo and black circles (●) indicate L-ornithine.

Changes in POMS scores are shown in Figure 1. There was a trend toward an improved mood indicated by each score compared to 0 weeks but there was no significant difference between the two groups. There was a significant improvement in self-reported “anger-hostility” at 2 weeks and 6 weeks in the L-ornithine group compared to the placebo group (Figure 1C).

### L-Ornithine supplementation improved sleep quality in study participants

**Figure 2:**
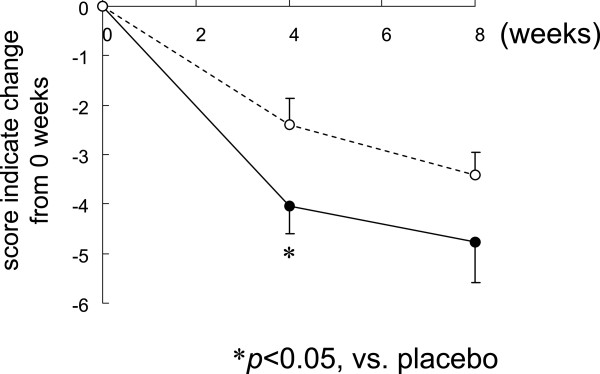
**Effect of L-ornithine supplementation on AIS.** Means of the change from 0 weeks of each AIS score to 4 and 8 weeks: mean ± SE. White circles (○) indicate the placebo and black circles (●) indicate L-ornithine.

**Figure 3:**
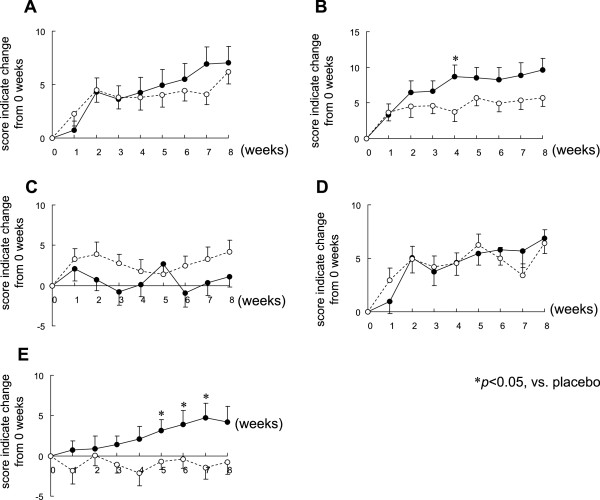
**Effect of L-ornithine supplementation on OSA.** Means of the change from 0 weeks of each OSA score (**A**, sleepiness on rising; **B**, initiation and maintenance of sleep; **C**, frequent dreaming; **D**, refreshing; **E**, sleep length) to 1, 2, 3, 4, 5, 6, 7 and 8 weeks: mean ± SE. White circles (○) indicate the placebo and black circles (●) indicate L-ornithine.

As shown in Figure 2, the AIS score revealed a trend towards improved insomnia in both groups from 0 weeks to 8 weeks. Furthermore, the AIS score indicated significant improvement in the L-ornithine group at 4 weeks in comparison with the placebo group.Scores for the OSA-MA for each group over an 8-week period are shown in Figure 3. Scores of three OSA-MA items, “sleepiness on rising” (Figure 3A), “initiation and maintenance of sleep” (Figure 3B) and “refreshing” (Figure 3D), tended towards improved sleep quality in both groups without significant between-group differences. Scores for “frequent dreaming” (Figure 3C) and “sleep length” (Figure 3E) were unchanged in the placebo group, however, the score for the L-ornithine group significantly improved for self-reported “initiation and maintenance of sleep” (Figure 3B) at 4 weeks and “sleep length” (Figure 3E) at 5 to 7 weeks in comparison with the placebo group.

### L-Ornithine supplementation attenuated the serum cortisol/DHEA-S molar ratio in study participants

**Figure 4:**
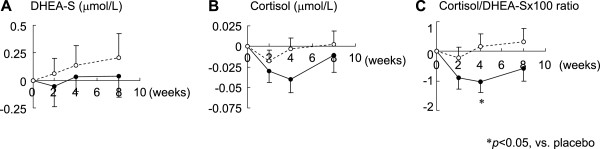
**Effect of L-ornithine supplementation on serum stress markers.** Means of the change from 0 weeks of each stress marker level (**A**, DHEA-S; **B**, cortisol; **C**, cortisol/DHEA-S) to 2, 4, and 8 weeks: mean ± SE. White circles (○) indicate the placebo and black circles (●) indicate L-ornithine.

While the concentration of serum DHEA-S was not increased by L-ornithine intake, serum cortisol concentration decreased in the L-ornithine group. Although the change in either level did not differ significantly between the two groups at any examination point (Figure 4A, 4B), the change in cortisol/DHEA-S ratio significantly decreased in the L-ornithine group after 4 weeks (Figure 4C). The cortisol/DHEA-S (×100) ratio was calculated based on serum cortisol and DHEA-S concentrations.

## Discussion

Our results showed that L-ornithine supplementation had a favourable effect on the cortisol response as an objective stress marker and improved perceived mood and sleep quality related to fatigue as well as subjective feelings derived from stress.

Previous studies reported that i.c.v. injections of L-ornithine had sedative and hypnotic effects on neonatal chicks exposed to acute stressful conditions [31,32]. That action was mediated by GABA_A_ receptors. It was also confirmed that orally administered L-ornithine can be transported into the brain of mice [20], and as a result, reduced the plasma corticosterone concentration induced by restraint stress in mice. L-ornithine levels in the brain increased after oral administration of L-ornithine in mice [20]. Moreover, it was shown that i.c.v. injected L-arginine, the precursor of L-ornithine, increased both L-arginine and L-ornithine concentrations in the telencephalon and diencephalon in chicks 10 min post-injection [33], however, the GABA content was not changed. This suggests that the sedative and hypnotic effects of L-ornithine were not due to changes in GABA synthesis [32].

The role of GABA in HPA axis regulation has been well established, indicating that corticotropin-releasing hormone (CHR) neurons receive robust GABAergic inhibition [34]. In addition, micro infusion of GABA agonists, such as the stress-derived neurosteroid tetrahydrodeoxycorticosterone (THDOC), into the paraventricular nucleus (PVN) decreased circulating levels of stress hormones [35]. Our result showing that orally administered L-ornithine decreased the serum cortisol level in human subjects was in agreement with previous animal studies on the effectiveness of stress reduction through the alleviation of HPA axis hyperactivity [20,31-34].

We observed that L-ornithine supplementation for 8 weeks reduced the serum cortisol level and cortisol/DHEA-S ratio, mainly due to reduction in the cortisol level. An imbalance between cortisol and DHEA-S may be a key factor in physical and psychiatric disease [36,37]. The molar DHEA-S/cortisol ratio was shown to be significantly lower in non-medicated depressed patients than in control subjects, and evening salivary DHEA/cortisol ratios were inversely correlated with the length of the current depressive episode [38]. Elevated cortisol/DHEA-S ratios in schizophrenia patients were positively associated with higher scores for anxiety and anger, depression and hostility, age, age at onset of illness, and duration of illness [39].

These reports provide support for our results showing that the decrease in cortisol/DHEA-S ratios through the administration of L-ornithine corresponded to the improvement in mood related to “anger-hostility” as well as sleep quality.

The initial decreasing tendency of the cortisol/DHEA-S ratios in the placebo group may be due to a non-specific, natural change which occurs after formal ornithine and placebo intake treatments. It is widely accepted that on average, brief initial interventions yield outcomes similar to those with prolonged treatments, suggesting that changes could be triggered after a brief phase of treatment [40,41].

“Anger-hostility”, an item on the POMS, is one of the phenotypes resulting from activation of sympathetic nerves. A physiological change accompanying stress was shown to be the increased excretion of cortisol and adrenaline [42]. Adrenalin promotes activation of the sympathetic nerve system suggesting L-ornithine supplementation might affect, not only the HPA axis, but also the autonomic nervous system. Unfortunately, we did not examine whether L-ornithine supplementation could affect either the autonomic nervous system or adrenaline levels.

Our results also suggested that sleep quality was improved by L-ornithine, as revealed by both AIS and OSA-MA questionnaires, along with alleviation of stress (cortisol/DHEA-S). The present finding is in accordance with a previous report that L-ornithine supplementation after alcohol consumption improved sleep quality as perceived upon awakening in flushers [43] and that ornithine increased the amount of non-rapid eye movement (NREM) sleep without reducing the power spectrum density of NREM sleep in mice [44]. This result replicated findings of our previous study, that ornithine could improve sleep in an animal model and suggested the effectiveness of orally administered L-ornithine on stress reduction through improvement of sleep quality in human subjects.

Furthermore, it was shown that L-ornithine administration stimulated release of growth hormone [45,46], which is secreted as the largest pulse after the onset of sleep, and that there is a correlation between night-time growth hormone release and sleep satisfaction [47-49].

A serotonin metabolite (5-hydroxyindole acetic acid, 5-HIAA) was induced in the striatum after L-ornithine supplementation [20]. Day-time serotonin levels stimulate production of melatonin during the night [50-52], therefore, L-ornithine might be considered an important nutrient to maintain the circadian rhythm and to allow individuals to sleep well.

Job stress is one of the most important social problems for workers today. This study has provided further objective evidence of the usefulness and effectiveness of L-ornithine for managing stress and sleep quality related to fatigue. Future studies must address how L-ornithine affects regulation of blood glucose and the autonomic nervous system.

Limitations exist in this study: first, we did not evaluate plasma ornithine levels, so we were unable to assess any direct correlation between ornithine and suppressing stress markers or improved subjective feelings. Second, we used three tests to assess fatigue and mood states, however, many tests, using differing strategies, are required to properly evaluate psychological status. Third, we determined the sample size of this study on the basis of a previous report [26], however, this sample size (n = 26) was not sufficient to lead to draw firm conclusions and more extensive studies are needed to confirm our findings.

## Conclusion

L-ornithine plays a central role in the urea cycle which converts ammonia to urea in the liver. L-ornithine administration has been known to enhance detoxification of ammonia in the liver. Recent reports have shown that L-ornithine has a positive effect on animal stress models. Our study suggested that L-ornithine has a positive effect on stress and sleep in healthy workers. L-ornithine might be beneficial for people who live a stressful life.

## Abbreviations

DHEA-S: Dehydroepiandrosterone-sulphate; DHEA: Dehydroepiandrosterone; POMS: Profile of Mood States; AIS: Athens Insomnia Scale; OSA-MA: Ogri-Shirakawa-Azumi sleep inventory MA version; HPA: Hypothalamic-pituitary-adrenal; GABA: Gamma-aminobutyric acid; CHR: Corticotropin-releasing hormone; THDOC: Tetrahydrodeoxycorticosterone; PVN: Paraventricular nucleus; NREM: Non-rapid eye movement; 5-HIAA: 5-hydroxyindole acetic acid.

## Competing interests

In this study, we used L-ornithine monohydrochloride, a product of Kyowa Hakko Bio Company, Limited. This company is an affiliate of Kirin Company, Limited, to which the authors belong. None of the authors had a personal or financial conflict of interest.

## Authors’ contributions

MM was involved in designing the trial, writing the trial protocol, calculating the sample size, analysing data, drafting and finalising the manuscript. TKo, TKi and YM were involved in designing the trial, writing the trial protocol, supervising analysis of data, and drafting and revising the manuscript. MK was involved in designing the trial. AT and HO supervised the trial as the principal investigator and participated in drafting and revising the manuscript. All authors contributed to the data interpretation and approved the final version of the manuscript.
